# Loading and Unloading Weaned Pigs: Effects of Bedding Types, Ramp Angle, and Bedding Moisture

**DOI:** 10.3390/ani4040742

**Published:** 2014-12-03

**Authors:** Arlene Garcia, John J. McGlone

**Affiliations:** Department of Animal and Food Sciences, Texas Tech University, Lubbock, TX 79409, USA; E-Mail: arlene.garcia@ttu.edu

**Keywords:** weaning pigs, ramp, slips, falls, vocalizations, animal welfare

## Abstract

**Simple Summary:**

Current guidelines suggest the use of ramps below 20° to load and unload pigs; they do not suggest the use of any specific bedding. Bedding types (nothing, feed, sand, wood shavings, and hay) were tested with four week old weaned pigs to determine which was most effective in reducing slips, falls, and vocalizations at three ramp angles, two moistures, over two seasons. Slips, falls, and vocalizations were summed to establish a scoring system to evaluate treatments. Scores increased in a linear fashion as ramp slope increased. The amount of time it took to load and unload pigs was affected by bedding type and ramp angle. Overall, the use of selected bedding types minimized slips, falls, and vocalizations and improved animal welfare.

**Abstract:**

The use of non-slip surfaces during loading and unloading of weaned pigs plays an important role in animal welfare and economics of the pork industry. Currently, the guidelines available only suggest the use of ramps below 20° to load and unload pigs. Three ramp angles (0°, 10° or 20°), five bedding materials (nothing, sand, feed, wood shavings or wheat straw hay), two moistures (dry or wet bedding; >50% moisture) over two seasons (>23.9 °C summer, <23.9 °C winter) were assessed for slips/falls/vocalizations (n = 6,000 pig observations). “Score” was calculated by the sum of slips, falls, and vocalizations. With the exception of using feed as a bedding, all beddings provided some protection against elevated slips, falls, and vocalizations (*P* < 0.01). Providing bedding reduced (*P* < 0.05) scores regardless of whether the bedding was dry or wet. Scores increased as the slope increased (*P* < 0.01). Provision of bedding, other than feed, at slopes greater than zero, decreased slips, falls and vocalizations. The total time it took to load and unload pigs was affected by bedding type, ramp angle, and season (*P* < 0.05). Minimizing slips, falls, and vocalizations when loading and unloading pigs improved animal welfare.

## 1. Introduction

The use of non-slip surfaces during loading and unloading of pigs plays an important role in animal welfare and economics of the pork industry. Losses equate to millions of dollars due to damaged/injured pigs; the incidence of transport losses is estimated to be about 1% of all pigs marketed [1,2]. Steep loading ramps have also been associated with injuries and prolapses [3,4]. For cattle, pigs, and sheep the maximum recommended angle for adjustable ramps is 25° and 20° for non-adjustable ramps [5]. Most experts would agree that anything below 20° is an acceptable ramp angle [6,7]. Currently, the guidelines available only suggest the use of ramps below 20° to load and unload pigs. However, these guidelines do not suggest the use of non-slip materials on the ramp floor. The use of non-slip materials could possibly improve welfare by minimizing slips, falls and vocalizations when loading and unloading pigs, as well as reduce financial loss caused by poor animal welfare.

Animal welfare is an important attribute in the concept of “food quality” [8], and there is an increasing consumer demand for higher animal welfare standards [9]. Producers, processors, retailers, and restaurants have added value to their products in response to the demand in animal welfare standards. However, for producers that do not integrate improved animal welfare conditions, new marketing schemes may result in a less profitable and low quality product [9].

The objective of this study was to investigate the effects of ramp angle, bedding material, moisture of bedding material, and season on welfare of finishing pigs. 

## 2. Experimental Section 

Pigs were PIC USA genetics using the Camborough-22 sow line and the 280 boar line. All animals were fed a diet to meet or exceed NRC nutrient requirements. Feed and water were provided ad libitum. All animal procedures were approved by the Texas Tech University Animal Care and Use Committee.

Three ramp angles (0°, 10° or 20°), 5 bedding materials nothing (N), sand (S), feed (F), wood shavings (WS) or wheat straw hay (H), and 2 moistures (dry or wet bedding or floor) over 2 seasons (>23.9 °C summer, <23.9 °C winter) were assessed for slips/falls and vocalizations on weaning barrows and gilts. The study included 1200 weaned pigs in a multifactorial design (5 beddings × 2 moistures × 3 ramp slopes × 2 seasons = 60 treatments). Pigs were handled in units of 20 pigs per group. Five, 20-pig replications of weaned pigs were evaluated per treatment. There were a total of 6,000 pig observations (20 animals/treatment × 60 treatments × 5 replications). Since the number of required animals was high for weaned piglets, each group of 20 piglets was used to evaluate no more than 10 randomly-selected treatments out of the 60 possible treatments. Weaned pigs that were injured, lame, or apparently sick were not used on the study.

The bedding material was either dry (greater than 80% dry matter with a target of 90% dry matter) or wet (less than 80% dry matter with a target of 50% dry matter). Seasons were determined by outside air temperature. Temperatures were categorized as summer (>23.9 °C to <37.8 °C) or winter (> −6.7 °C to <23.9 °C). Air temperature was used as a covariate within season in the statistical model, as well as air temperature effects. Temperature and wind speed outside the building were recorded every 5 min using a Kestrel^®^ 4500 (Nielsen-Kellerman, Boothwyn, PA, USA).When bedding was used on the ramp, it covered all the floor surface of the ramp. When wood shavings and straw were used as bedding material, its depth was 9.5 mm which is equivalent to using 1 bale (22.7 kg) of wood shavings in a 1.3 m × 2.5 m ramp. Similarly, when feed (non-pelleted, ground mixture of corn and soybean meal) and sand were used, 6.5 mm depth of bedding was used to cover the entire ramp floor surface. Twenty weaned pigs were loaded at a time and all the measures were recorded using a digital Sony^®^ camcorders DCR-SR85 (Sony, San Diego, CA, USA). A camcorder was fitted at the back of the trailer facing towards the exit door of the barn (loading) and another was placed in the building facing towards the ramp (unloading) to record slips, falls and vocalizations. Only 2 trained personnel were involved in moving the pigs and observing the video.

Barrows and gilts were weaned at 21 ± 3 d and put into groups of 20 pigs (4.5–6.8 kg) per pen on wire floored pens (2.4 m × 1.2 m). One week after weaning, 1 pen of 20 pigs was removed from their home pen and walked a distance of 37.5–46.7 m inside the building with a 1.2 m wide aisle and walked onto a ramp with the random treatment and onto a trailer. The ramp had a metallic chute, with a total length of 3.6 m and adjustable height. The chute was solid on the sides to 0.9 m high, then partially open above 0.9 m above the solid side. The ramp had cleats 0.3 m apart to prevent slips and falls. When pigs were reluctant to move, a high pitch whistling sound was made, or sorting board was used. Pigs walked on the ramp for a length of 4.6 m and then into the trailer. Pigs were moved the same distance irrespectively of ramp angle to get inside the trailer. Trailer pens were 2.1 m × 2.4 m dimension. Pigs remained on the trailer for 30 min then were unloaded from the trailer, down the ramp with the same treatment and returned to their home pen. Digital camcorders were placed so that the first and last steps on and off the ramp were recorded in order to determine the total time to load/unload. The time it took to load and unload the pigs was determined by the first pig’s step onto the ramp and ended when the last pig stepped off the ramp onto the trailer (loading) or onto the aisle (unloading). The loading and unloading times were added to determine the total time. Video was analyzed for slips, falls, and vocalizations.

The sum of slips, falls, and vocalizations were recorded as a score in part because the data set for any one measure contained many zero values. Treatments were then given a score based on the observations. As the slips, falls, and vocalizations increased, the score increased. Lower scores meant a lower number of slips, falls, and vocalizations which was considered better than high scores. Slips were defined as when one foot missed a step but the pig caught itself; falling was considered an imbalance of the pig’s body with some part of the body physically touching the floor; vocalizations were any squeals produced by the pigs other than grunts. 

The study used a Complete Randomized Design with 5 repetitions per treatment examining a total of 60 treatments. All data were analyzed using SAS 9.3 General Linear Models procedure (SAS, 2010 SAS Inst., Inc., Cary, NC, USA). Data were analyzed using analysis of variance procedures in SAS. The statistical model included the effects of bedding, slope, wet/dry, season, all possible interactions, and temperature as a covariate. When wind was used as a covariate it was not significant. All data were tested for homogenous variances and normal distributions. The experimental unit was a group of 20 pigs. 

## 3. Results and Discussion

Bedding types (nothing, feed, sand, wood shavings, and hay) were used on a ramp to determine which was more effective in preventing slips, falls, and vocalizations at different angles (0°, 10°, 20°), moisture levels (wet or dry) and seasons (summer or winter). Presented in Table 1 are *P* values for each measure over each variable in the model. The score combines each of the measures. Because so many observations were zero (ex. no slips, falls or vocalizations at 0° slope), the score may be the most robust measure. A combined view of score and total time (TTime) to load and unload gives the best overall view of the results. Main effects will be summarized first followed by interactions. 

### 3.1. Bedding Effect

**Figure 1:**
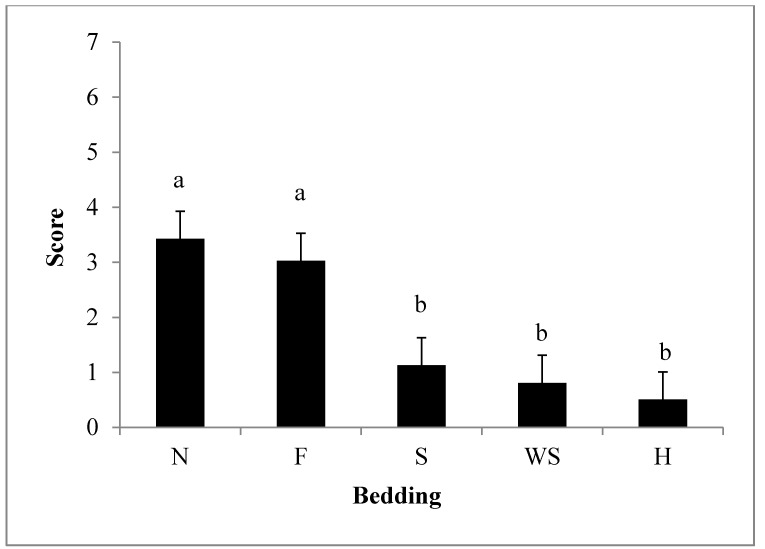
Least Squares means ± 0.53 for weaned pig scores with the use of different types of bedding materials (*P* < 0.01). Beddings abbreviated by N = nothing, F = feed, S = sand, WS = wood shavings, H = hay. Bedding was rated based on a score system, which was calculated by the sum of slips, falls, and vocalizations. n = 60 observations/bedding types.

With the exception of using feed as bedding, all bedding materials provided some protection against a higher levels of scores (*P* < 0.01) compared to no bedding (Table 1). Score levels were lower for bedding that provided the most protection (Figure 1). 

**Table 1:** Least Squares means accompanied by pooled standard error for slips, falls, vocalizations, time (sec), total time (TTime: sec) and score for weaned pigs during loading (U) and unloading (D) with the use of different bedding materials.

Measure	Nothing	Feed	Sand	Shavings	Hay	Se	*P* value
**Slip (u)**	1.4 ^a^	0.7 ^b^	0.2 ^bc^	0.4 ^bc^	0.1 ^c^	0.2	0.0023 *
**Fall (u)**	0.4 ^a^	0.4 ^ab^	0.2 ^bc^	0.1^c^	0.1 ^c^	0.1	0.0023 *
**Vocal (u)**	0.8	1.1	0	0.2	0.0	0.4	0.5268
**Time, s (u)**	91.3	92.1	82.2	87	65.2	9.6	0.1260
**Slip (d)**	0.5 ^a^	0.6 ^a^	0.4 ^a^	0.1 ^b^	0.1 ^b^	0.1	0.0001 *
**Fall (d)**	0.2	0.2	0.2	0	0.2	0.1	0.1372
**Vocal (d)**	0.1	0.1	0.1	0	0	0.1	0.2110
**Time, s (d)**	34.6 ^a^	34.4 ^a^	19.3 ^b^	29.2 ^ac^	25 ^bc^	3.2	0.0010 *
**TTime, s**	126	126	101	116	90.1	11.2	0.0780
**Score**	3.4 ^a^	3.0 ^a^	1.1 ^b^	0.8 ^b^	0.5 ^b^	0.5	0.0086 *

^a–c^ Within a row, means without a common superscript differ (*P* < 0.05). * *P* < 0.05.

Least Squares means accompanied by a different superscript differs (*P* < 0.05).

### 3.2. Moisture Effects

**Figure 2:**
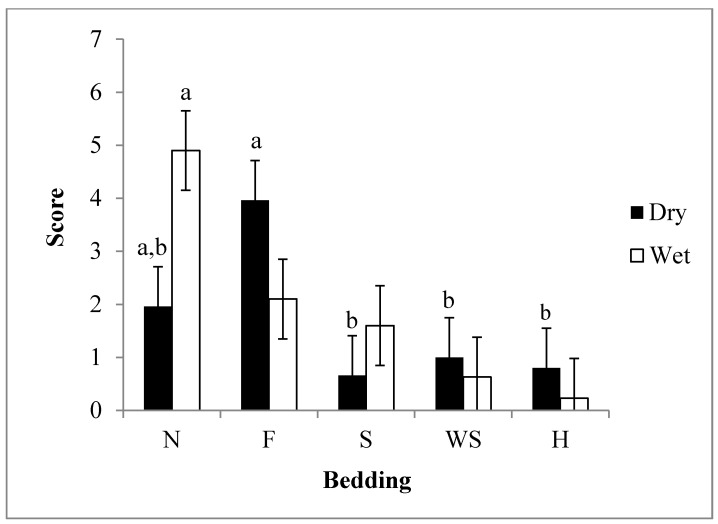
Least Square means ± 0.33 for weaned pig scores with the use of wet or dry bedding materials (*P* < 0.05). Beddings abbreviated by N = nothing, F = feed, S = sand, WS = wood shavings, H = hay. Bedding was rated based on a score system which was calculated by the sum of slips, falls, and vocalizations. n = 30 observations/bedding moisture.

The interaction between moisture and bedding type was significant at (*P* < 0.05). The scores for dry ramp within bedding were similar, with the exception of feed (Figure 2). Scores for nothing were not different than the other beddings, including feed (*P* > 0.05), but dry feed scores differed from other beddings (*P* < 0.05). The most evident protection on a dry surface was provided respectively by sand, hay, and wood shavings. The lowest score on a dry surface was with sand (0.6 ± 0.75; *P* > 0.05). On a wet surface the use of hay, wood shavings, sand, and feed reduced scores significantly compared to nothing. The lowest score with a wet surface was with hay (0.8 ± 0.75; *P* > 0.05). In the current study, using feed as a bedding was not beneficial in reducing slips, falls, and vocalizations when the ramp was dry. However, if the ramp surface was wet, using feed, or any other bedding was better than not using anything at all.

Least Squares means accompanied by a different superscript differs (*P* < 0.05).

**Figure 3:**
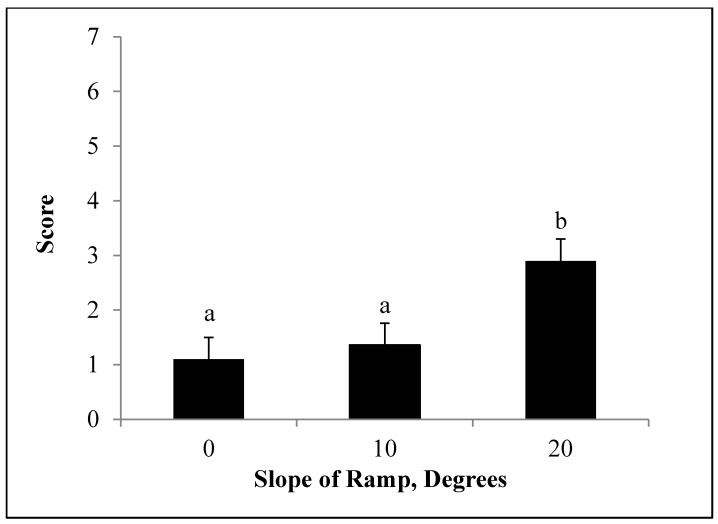
Least Squares means for weaned pig scores at different ramp slopes (*P* < 0.01). Scores were calculated based on the sum of slips, falls, and vocalizations. n = 300 observation of 20 pigs each.

Least Squares means accompanied by a different superscript differs (*P* < 0.05).

### 3.3. Slope Effect

Scores increased with increasing slopes (Figure 3). A ramp with a 20° slope caused a higher score (*P* < 0.05) than either 0 or 10° slopes. Scores increase by double from 0° to a 10° slope and almost by triple from 0° to a 20° slope. Therefore, the linear increase in scores suggest it is more effective to use a lower slope to decrease scores, but if decreasing the slope is not a possibility the use of bedding is beneficial.

### 3.4. Interactions

**Figure 4:**
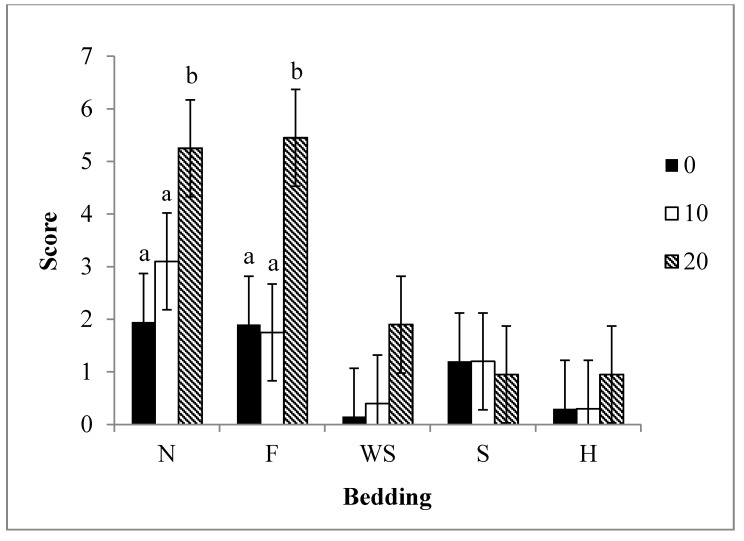
Least Squares means ± 0.92 for weaned pig scores at different ramp slopes with the use of different bedding materials (*P* < 0.01). Beddings abbreviated by N = nothing, F = feed, S = sand, WS = wood shavings, H = hay. Bedding was rated on a score system which was calculated by the sum of slips, falls, and vocalizations. n = 12 observations/bedding type.

The bedding by slope effect was significant (*P* = 0.01; Figure 4). There was no difference in scores between 0° to 10° slopes for all bedding types. The use of nothing and feed as a bedding had higher scores at a 20° slope than at lower slopes. Additionally, nothing and feed had higher scores at a 20° slope than all other bedding types used (*P* < 0.05). The use of wood shavings, sand, and hay showed to decrease scores regardless of the slope of the ramp. 

Least Squares means accompanied by a different superscript differs (*P* < 0.05).

There was a significant 3-way interaction for moisture level, season, and slope for score (*P* < 0.05). Scores for 20° slopes were higher than the other slopes among season and moisture levels (Table 2).

**Table 2:** Least Squares means ± 0.82 for weaned pig scores in response to the 3-way interaction for moisture (D = Dry, W = Wet), season, and slope (*P* < 0.05). Score was calculated by the sum of slips, falls, and vocalizations. n = 60 treatments.

Moisture	Season	Slope	Score
**D**	Summer	0	0.8
**D**	Summer	10	1.4
**D**	Summer	20	1.6
**D**	Winter	0	0.8 ^a^
**D**	Winter	10	0.6 ^a^
**D**	Winter	20	4.7 ^b^
**W**	Summer	0	0.9 ^a^
**W**	Summer	10	1.4 ^a^
**W**	Summer	20	3.0 ^b^
**W**	Winter	0	1.7
**W**	Winter	10	1.9
**W**	Winter	20	2.2

Least Squares means accompanied by a different superscript within each moisture and season differ (*P* < 0.05).

The 20° slope for a dry surface during winter and a wet surface during summer had the highest scores when compared to other beddings, moisture levels, and seasons. The longest total times for loading and unloading was found when no bedding was used at a 20° slope during summer on a wet surface (Table 3), and the fastest time was when hay was used at a 10° slope during winter on a wet surface (Table 4). Additionally, an additive effect was seen with certain beddings. If the ramp was at a 20° slope and had no bedding on it and the surface was wet, scores increased, and the time it took to load also increased. 

During the course of the study, it was observed that certain beddings were effective in decreasing scores but also increased total times, possibly due to the pigs being distracted by the bedding. This behavior was observed mostly with wet wood shavings, which distracted the pigs and caused them to spend more time playing and eating the bedding than going up the ramp. The amount of time spent loading and unloading is important in the swine industry since loading pigs is considered a critical part of the transport stage. The delay in loading and unloading due to unmanageable pigs may be frustrating to the handler, and even small amounts of threatening behaviors by humans can produce a chronic stress response in pigs [10,11]. Both cattle and pigs remember bad experiences and when handled roughly they are harder to handle in the future [12,13]. Pigs are socially investigative (investigate con-specifics) or non-socially investigative (investigate the environment) [14] either the smell or the consistency of the bedding in the current study may have caused the pigs to increase exploring; thus slower traffic up the ramp may reduce slips, falls, and vocalizations while increased total time. 

**Table 3:** Least Squares means ± 39.09 for total time (TTime: sec) spent loading and unloading weaned pigs during the summer in response to the four-way interaction of bedding, moisture level (W/D), season, and slope (*P* < 0.05). Score was calculated by the sum of slips, falls, and vocalizations. n = 60 treatments.

Bedding	Moisture level ^1^	Season	Slope, °	TTime, sec	Score
**Nothing**	D	Summer	0	112.2	0.6
**Nothing**	D	Summer	10	113.2	1.2
**Nothing**	D	Summer	20	78.2	1.4
**Nothing**	W	Summer	0	75.2 ^a^	1.4
**Nothing**	W	Summer	10	133.6 ^a^	4.4
**Nothing**	W	Summer	20	257.2 ^b^	11.2
**Feed**	D	Summer	0	191.2 ^a^	2.8
**Feed**	D	Summer	10	83.0 ^b^	4.8
**Feed**	D	Summer	20	121.6 ^a,b^	4.0
**Feed**	W	Summer	0	122.2	1.4
**Feed**	W	Summer	10	101.0	1.0
**Feed**	W	Summer	20	99.0	2.4
**Sand**	D	Summer	0	137.4	0.4
**Sand**	D	Summer	10	82.2	1.0
**Sand**	D	Summer	20	143.0	0.8
**Sand**	W	Summer	0	101.4	1.4
**Sand**	W	Summer	10	105.6	0.6
**Sand**	W	Summer	20	60.8	1.4
**Shavings**	D	Summer	0	96.2	0.0
**Shavings**	D	Summer	10	121.4	0.0
**Shavings**	D	Summer	20	119.0	0.2
**Shavings**	W	Summer	0	99.4	0.2
**Shavings**	W	Summer	10	164.8	1.2
**Shavings**	W	Summer	20	109.0	0.0
**Hay**	D	Summer	0	104.6	0.4
**Hay**	D	Summer	10	116.6	0.2
**Hay**	D	Summer	20	73.8	1.6
**Hay**	W	Summer	0	138	0.4
**Hay**	W	Summer	10	110.8	0.2
**Hay**	W	Summer	20	79.4	0.2

Least Squares means accompanied by a different superscript differ (*P* < 0.05) within a bedding, moisture level, and season. ^1^ Moisture levels: dry (D); wet (W).

**Table 4:** Least Squares means ± 39.09 for total time (TTime: sec) spent loading and unloading weaned pigs during the winter in response to the four-way interaction of bedding, moisture level (W/D), season, and slope (*P* < 0.05). Score was calculated by the sum of slips, falls, and vocalizations. n = 60 treatments.

Bedding	Moisture level ^1^	Season	Slope, °	TTime, sec	Score
**Nothing**	D	Winter	0	82.4 ^a^	2.0
**Nothing**	D	Winter	10	112.8 ^a^	0.8
**Nothing**	D	Winter	20	223.4 ^b^	5.8
**Nothing**	W	Winter	0	105.8	3.8
**Nothing**	W	Winter	10	136.6	6.0
**Nothing**	W	Winter	20	81.6	2.6
**Feed**	D	Winter	0	73.2	1.4
**Feed**	D	Winter	10	103.8	0.0
**Feed**	D	Winter	20	170.6	10.8
**Feed**	W	Winter	0	251 ^a^	2.0
**Feed**	W	Winter	10	131.8 ^b^	1.2
**Feed**	W	Winter	20	70.8 ^b^	4.6
**Sand**	D	Winter	0	124.2	0.2
**Sand**	D	Winter	10	97.8	1.2
**Sand**	D	Winter	20	85.2	0.4
**Sand**	W	Winter	0	116.8	2.8
**Sand**	W	Winter	10	83.8	2.2
**Sand**	W	Winter	20	80.4	1.2
**Shavings**	D	Winter	0	91.6 ^a^	0.2
**Shavings**	D	Winter	10	67.0 ^a^	0.4
**Shavings**	D	Winter	20	217.6 ^b^	5.2
**Shavings**	W	Winter	0	110.2 ^ab^	0.2
**Shavings**	W	Winter	10	188.0 ^a^	0.0
**Shavings**	W	Winter	20	73.8 ^b^	2.2
**Hay**	D	Winter	0	114.8	0.4
**Hay**	D	Winter	10	75.4	0.6
**Hay**	D	Winter	20	65.2	1.6
**Hay**	W	Winter	0	90.8	0.0
**Hay**	W	Winter	10	46.8	0.2
**Hay**	W	Winter	20	65.4	0.4

Least Squares means accompanied by a different superscript differ (*P* < 0.05) within a bedding, moisture level, and season. ^1^ Moisture levels: dry (D); wet (W).

Some of the delays in loading or unloading may not directly be caused by bedding. Other than investigative behaviors, an animal’s aversion to a situation can increase loading and unloading times. Aversion to a situation may be characterized by freezing, not moving forward, backing up, running away, or vocalizing [15]. It has also been suggested that pigs refuse to load when it is either too cold or too bright outside [16,17]. Therefore, the current study shows that several factors should be considered in combination to identify the appropriate bedding for the specific occasion.

## 4. Conclusions

The use of some type of bedding when loading and unloading pigs on a ramp is beneficial in reducing slips, falls, and vocalizations; whereas, not using any bedding may increase the occurrence of these. To our knowledge, the type of bedding to be used on ramps to reduce slips, falls, and vocalizations during loading and unloading has not been evaluated. In most occurrences, if bedding is used at all, the choice of material is based on what is cheapest or what may be at hand. 

As the slope of the ramp increased scores increased. Therefore, the linear increase in scores suggests it is more effective to use a lower slope to decrease scores and if decreasing the slope is not a possibility the use of bedding is beneficial. Additionally, some bedding types may increase total times to load and unload weaned pigs. Therefore, several factors should be considered in combination to identify the appropriate bedding for the specific occasion. 

Further studies are needed to find more effective non-slip footing surfaces. Cleats spaced to the length of the pigs stride can prevent leg injuries [18]. However, when cleats are too close together the animal will step on top of the cleats instead of between them, not providing traction [19]. If the cleats are spaced too far apart then they can also cause slipping and possibly damage piglet dew claws [19]. This is because most ramps are made for finishing pigs or cattle and, therefore are not appropriate for weaned pigs. Stair step ramps on concrete have also been reported to be effective non-slip footing surfaces [20,21], but concrete reinforcing rods can also make good cleats on steel ramps and provide a good non-slip surface as long as cleats are adequately spaced [19]. The use of rubber tire mats also may merit further research. Rubber tire mats are economical and can be an effective non-slip surface. Providing non slip surfaces is of the essence in order to stay compliant with animal welfare perspectives and to avoid monetary losses. Furthermore, there is a growing consensus toward the implementation of higher animal welfare standards. Vehicles used to haul animals, scales, and stunning areas should also consist of non-slip flooring [22].

Scientific data on material type, moisture of bedding, and ramp angles based on pigs’ size will allow pork producers to improve animal welfare quality, while also addressing financial cost of pre-slaughter handling. 
